# False Atrial Fibrillation Alerts from Smartwatches are Associated with Decreased Perceived Physical Well-being and Confidence in Chronic Symptoms Management

**DOI:** 10.26502/fccm.92920314

**Published:** 2023-04-03

**Authors:** Khanh-Van Tran, Andreas Filippaios, Kamran Noorishirazi, Eric Ding, Dong Han, Fahimeh Mohagheghian, Qiying Dai, Jordy Mehawej, Ziyue Wang, Darleen Lessard, Edith Mensah Otabil, Alex Hamel, Tenes Paul, Matthew F Gottbrecht, Timothy P Fitzgibbons, Jane Saczynski, Ki H Chon, David D McManus

**Affiliations:** 1 Division of Cardiovascular Medicine, Department of Medicine, University of Massachusetts, Chan Medical School, 55 Lake Avenue North, Worcester, MA 01655, USA; 2 Department of Population and Quantitative Health Sciences, University of Massachusetts Chan Medical School, 55 Lake Avenue North, Worcester, MA 01655, USA; 3 Department of Biomedical Engineering, University of Connecticut, 260 Glenbrook Road, Storrs, CT 06269, USA; 4 Division of Cardiovascular Medicine, Department of Medicine, Saint Vincent Hospital, 123 Summer Street, Worcester, MA 01608, USA; 5 Department of Pharmacy and Health Systems Sciences, Northeastern University, Boston, Massachusetts, USA

**Keywords:** AF, False AF Alerts, Pulsewatch Study

## Abstract

Wrist-based wearables have been FDA approved for AF detection. However, the health behavior impact of false AF alerts from wearables on older patients at high risk for AF are not known. In this work, we analyzed data from the Pulsewatch (NCT03761394) study, which randomized patients (≥50 years) with history of stroke or transient ischemic attack to wear a patch monitor and a smartwatch linked to a smartphone running the Pulsewatch application vs to only the cardiac patch monitor over 14 days. At baseline and 14 days, participants completed validated instruments to assess for anxiety, patient activation, perceived mental and physical health, chronic symptom management self-efficacy, and medicine adherence. We employed linear regression to examine associations between false AF alerts with change in patient-reported outcomes. Receipt of false AF alerts was related to a dose-dependent decline in self-perceived physical health and levels of disease self-management. We developed a novel convolutional denoising autoencoder (CDA) to remove motion and noise artifacts in photoplethysmography (PPG) segments to optimize AF detection, which substantially reduced the number of false alerts. A promising approach to avoid negative impact of false alerts is to employ artificial intelligence driven algorithms to improve accuracy.

## 1. Introduction

Early detection of atrial fibrillation (AF) can prevent the devastating consequences of strokes [1–3]. Nearly 20% of those who suffer ischemic strokes associated with AF are first diagnosed with the arrhythmia at the time of the stroke or soon after, highlighting the need for timely AF detection [4,5]. The American Heart Association recommends heart rhythm monitoring for undiagnosed AF in patients who suffered an embolic stroke of undetermined source [6,7]. Non-invasive adhesive monitors, such as the Zio^®^ Patch, Cardiac Insight patch, or mobile cardiac outpatient telemetry (MCOTTM) devices are often prescribed for this purpose [8–10]. The typical wear time for Zio^®^ Patch or Body Guardian is 14 days, whereas MCOTTM devices can be used for up to 30 days of monitoring. Implantable cardiac monitors (ICM), which provide AF monitoring over years, have been shown to detect higher rates of AF than conventional monitoring in the 12- months after a cryptogenic stroke, suggesting that prolonged monitoring after stroke can lead to higher rates of AF detection [11]. Despite the longer duration of surveillance with ICM, the higher cost and invasiveness of these devices limit their broad adoption [12]. The Pew Research Center reports one-in-five U.S. adults in their daily lives wear smartwatch with a fitness tracker. Many such devices are now capable of AF detection [13]. Several commercially available smartwatches, including from Apple, Samsung, and Fitbit, have been shown to be highly sensitive and specific for AF detection and have both pulse plethysmographic and electrocardiographic algorithms capable of detecting rhythm irregularity that are cleared by the FDA for incident AF detection [14–17]. To date, studies exploring smartwatches’ ability to detect AF include lower-risk populations, predominantly young smartwatch owners [18]. The usability and accuracy of wrist-based wearables to identify AF in populations at high risk for arrhythmias, including older stroke survivors, have not been thoroughly evaluated [19]. Older adults report less familiarity with digital health technology and thus maybe particularly impacted by notifications from smartwatches prescribed for AF monitoring. The Apple Heart study demonstrated that of the 450 participants who received notifications for irregular pulse, only 34 % had AF diagnosed with subsequent ECG patch monitor [18]. We hypothesize that false AF alerts may adversely impact quality of life and patient self-perceived well-being. We explore this hypothesis by analyzing data from the Pulsewatch study, a randomized clinical trial (NCT03761394) that examined the accuracy and acceptability of a smartwatch-smartphone app dyad for AF detection among stroke survivors [20].

## 2. Methods

### 2.1 Study Design and Population

We analyzed data from the Pulsewatch study, a randomized controlled trial in which participants were randomized 3:1 into intervention and control groups. In Phase I of the study, the intervention group was randomized to use the Pulsewatch system: an Android OS smartwatch-smartphone app dyad (Samsung Gear S3 or Samsung Galaxy Watch 3) capable of AF detection and wear a standard-of-care ECG patch monitor (Cardea Solo, Cardiac Insight, Seattle WA) for 14 days [20,21]. The control group was asked to wear the ECG patch monitor but did not receive a study smartwatch-smartphone dyad. The second phase of the Pulsewatch study focused on adherence to the smartwatch-smartphone app dyad and did not include contemporaneous patch monitoring or AF adjudication. As the focus of the present analysis was on the impact of false positive alerts, defined as alerts not triggered by AF as determined by ECG patch monitor and cardiologists, we used only information gathered from the intervention group from Phase I [20]. Eligible participants were recruited from neurology and cardiology ambulatory clinics at UMass Memorial Health from 2018–2021. Participants had to (1) be over 50 years old, (2) have had an ischemic stroke in the last decade, and (3) be willing to use the Pulsewatch system (smartwatch-smartphone app dyad) and undergo patch monitoring over the course of the study. Exclusion criteria included having contraindications to long-term anticoagulation, inability toprovide informed consent, contraindication for wearing an ECG patch monitor (e.g., sensitivity or allergy to medical adhesives, implantable pacemaker), or having a life-threatening arrhythmia that required immediate analysis and in-patient monitoring. The methods were performed in accordance with relevant guidelines and regulations and approved by UMass Chan Institutional Review Board (H00009953).

### 2.2 Study Procedures

**Figure 1:**
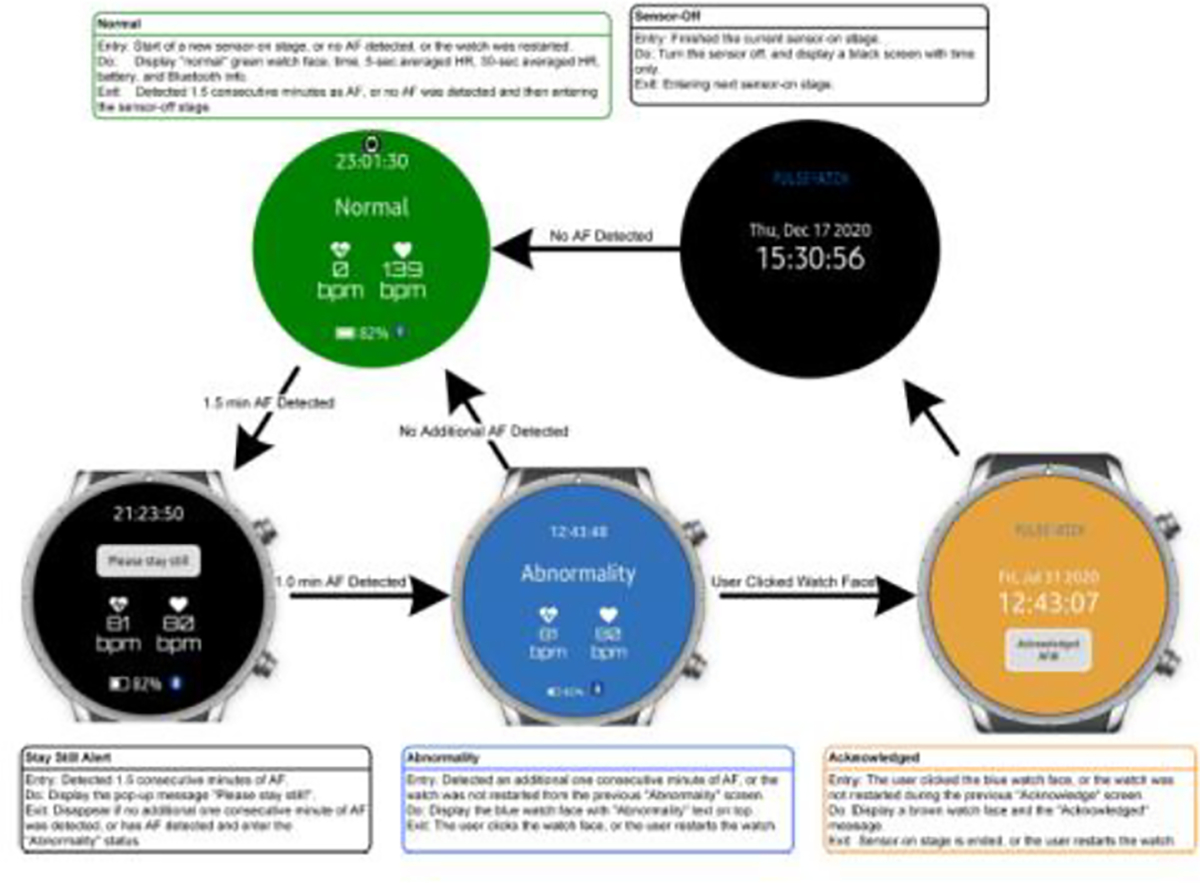
AF Alerts from our Pulsewatch smartwatch.

Eligible patients were identified through review of the electronic medical record. Once identified, an invitation letter was mailed to briefly describe the study, including a study number to call if they had additional questions or wanted to opt out of further contact. These potential participants were then approached at the time of their clinic appointment. If they chose to enroll in the study, they were provided written informed consent and filled out a baseline study questionnaire assessing several sociodemographic and psychosocial domains. At baseline and 14 days, participants completed validated questionnaires, including the generalized anxiety disorder-7 (GAD-7) scale, consumer health activation index (CHAI), the SF-12 (physical and mental health) survey, and the general disease management scale [22–25]. All participants were given a comprehensive set of instructions about proper watch and patch monitor use as well as in-person training at enrollment on operation of all study devices. Participants who were randomized to use the Pulsewatch system were coached to wear the watch as much as possible and to engage with the Pulsewatch phone app to log symptoms or review their data (including any alerts). Since the first phase of the study focused on accuracy, the study staff called intervention participants on the third and seventh days of the study to encourage watch wear and help troubleshoot any technical challenges they might experience. In our study, participants wore the Samsung smartwatch with PPG sensor program that ran every 10 min [26]. The duration of the sensor-on stage was 5 min, but it could be extended based on PPG findings (Figure 1). During the sensor-on stage, should there be 1.5 min of AF detected, participants were asked to “please hold still” for further 1.0 min analysis. “Abnormality” alert appeared if AF was detected in the 1.0 monitoring period. In our study, we considered any participant with both a “Please stay still” and “Abnormality” notification as having an AF alert. Smartwatch alerts for AF that did not correspond with AF identified on their Cardiac Insight ECG patch and cardiologist overread were considered to be false alerts. Sociodemographic, psychosocial, and clinical characteristics were compared between participants who received false alerts and those who did not. Chi-square tests and t-tests were used to examine between group differences for continuous variables. We used linear regression models to examine the association between those with and without false alerts, at baseline, and changes in anxiety, patient activation, and self-rated physical and mental health status, over a 14- day period. We did not adjust for confounding variables due to our modest sample size. P-value < 0.05 was considered to be statistically significant and all statistical analyses were completed using SAS 9.3.

### 2.3 Artificial Intelligence Driven Algorithms to Improve Accuracy

To increase data usability for AF detection, we propose reconstruction of artifact removed PPG segments using convolutional denoising autoencoder (CDA) [27]. Most prior studies typically used only one CDA for removing motion and noise artifact (MNA) from ECG with NSR [28]. Moreover, some studies have used CDA trained on NSR to remove MNA on data with AF. More recently, CDA was trained using equal number of AF and NSR data, but both approaches resulted in sub-optimal performances [29,30]. Our approach differs from prior CDA approaches in that we employ two distinct CDA models that were trained on either data with AF or data without AF (“non-AF”). In order to know which model to use on a segment with moderate MNA, however, we first had to classify the segment as either AF or non-AF using a deep learning (DL)-based classifier so that the appropriately trained CDA model for either AF or non-AF could be used to remove motion and noise artifact. The rationale for using two separate CDA models for either AF or non-AF is that, based on our investigation, we found that a specific CDA model trained for AF performed better on segments with AF than did a model that combined both AF and non-AF rhythms. Likewise, a specific CDA model trained for non-AF performed better on non-AF segments.

## 3. Results

**Figure 2:**
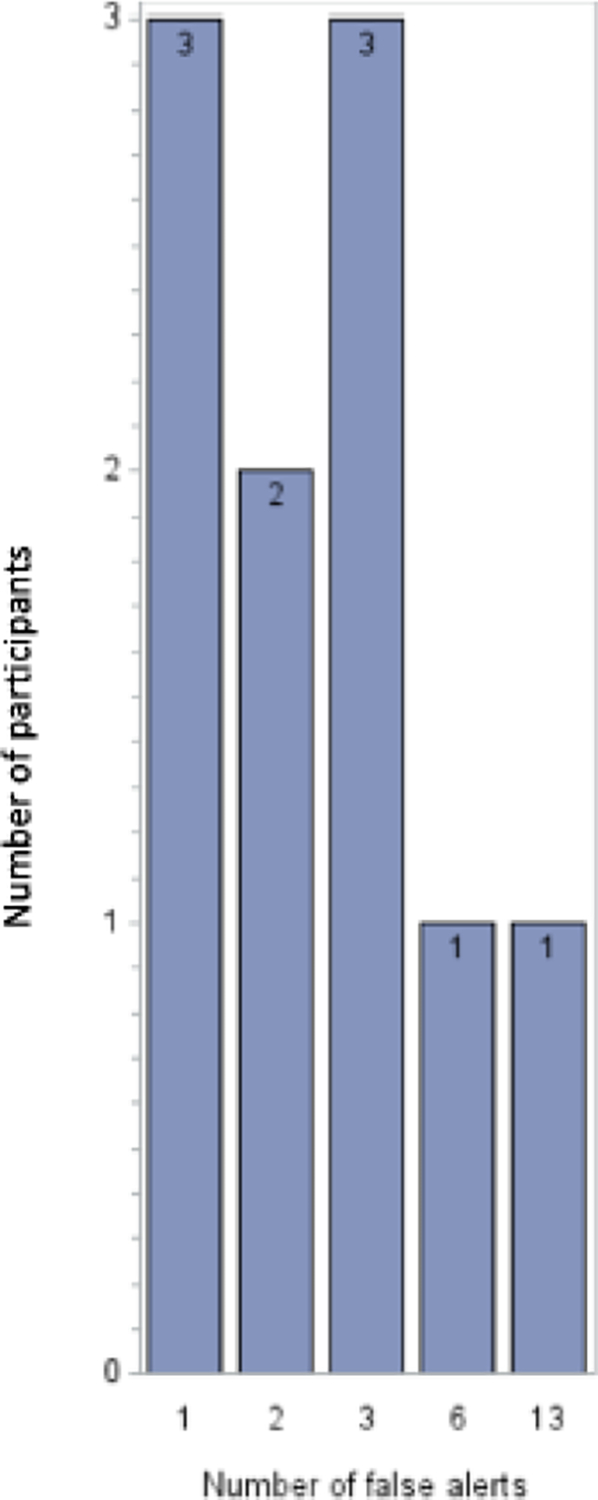
Distribution on false alerts received by participants.

**Figure 3:**
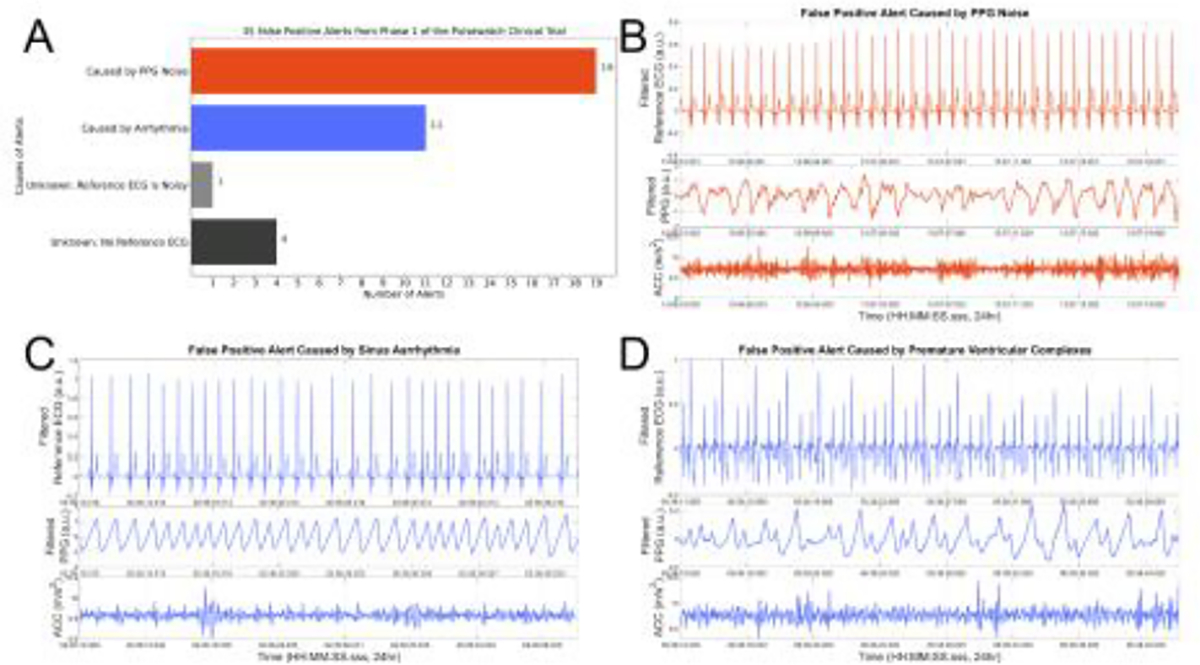
Underlying causes of false alerts (A) are found to be related to noise (B) and arrhythmia such as sinus arrhythmia (C) or premature ventricular complexes (D).

Of the eighty-five participants who were randomized to receive smartwatch-smartphone dyads, 15 received AF alerts from Samsung smartwatches (Supplemental Table 1–3). Ten of the 15 participants (67%) who received alerts did not have AF detected on a contemporaneous ECG patch monitor. The average age of the 80 participants (10 who received false alerts and 70 who received no alerts) in our study was 64.3 ± 9.1 years old. Eighty-six percent were white and 43% were female. Ten out of eighty participants (12%) had false AF alerts. Five participants received ≤ 2 alerts and five participants received >2 alerts, with the most being 13 false alerts received by one participant (Figure 2). Of the total 35 false alerts that occurred, 19 had underlying sinus rhythm with noisy PPG signals, and 11 were caused by arrhythmias, such as premature atrial or ventricular contractions as well as sinus arrhythmia. (Figure 3). There were 5 alerts that we were not able to determine underlying cause as ECG data was corrupted and not available. There were no significant differences in the baseline characteristics of those with false alerts as compared with those free from false alerts during the 14-day follow-up period (Table 1). Baseline blood pressure, heart rate, and BMI were similar among both groups. Additionally, there were no differences in psychosocial characteristics or technology engagement characteristics between the two groups (Table 2).

### 3.1 Change in Patient Reported Outcomes during the 14-day follow-up Period

Participants who received false AF alerts did not have statistically significant change in self-reported anxiety (GAD-7, β = −1.11 (1.28), P = 0.39, Table 3), patient activation (CHAI, β = −6.04 (4.14), P = 0.15, Table 3), or medication adherence (β = −1.06 (0.99), P = 0.29, Table 3) compared to those not exposed to false alerts over the study period. Similarly, participants with a false positive alert did not experience a significant decline in mental health compared to those free from an alert (β = 1.14 (2.65), P = 0.67, Table 3). Notably, participants with a false AF alert reported a statistically significant decrease in self-reported physical health (β = −7.53 (3.20), P = <0.02, Table 3) compared to those free from an alert over the study period. Furthermore, those who received >2 alerts reported a more significant decrease in self-reported physical health than did those who received ≤ 2 alerts (β = −14.08 (4.24), P = 0.001 vs β = −0.99 (4.24), P = 0.82, respectively, Table 4). Participants who received a false AF alert also reported less confidence in symptom self-management than did those who did not receive a false AF alert (β = −8.32 (2.81), P = 0.004, Table 3). Those participants who received > 2 alerts reported a greater decrease in their confidence in symptom self-management than did those who received ≤ 2 alerts (β = −12.32 (3.80), P = 0.002 vs β = −4.32 (3.80), P = 0.26, respectively, Table 4). Of note, we did not observe a statistically significant impact of true positive AF alerts on patient-reported outcomes, though this could be due to our modest sample size (Supplemental Tables 4–6).

### 3.2 Artificial Intelligence Driven Algorithms to Improve Accuracy

**Figure 4:**
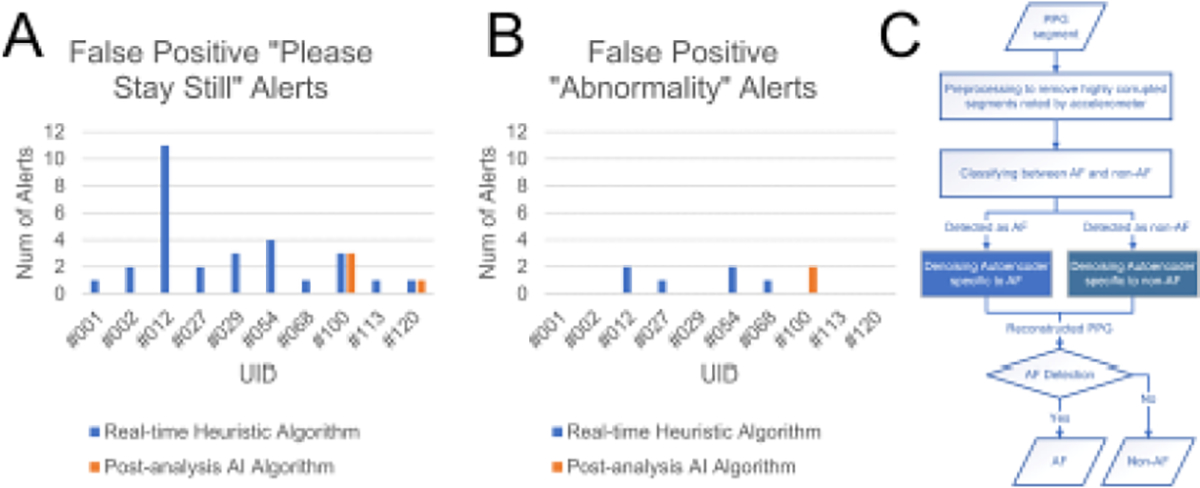
Comparison of the number of false positive alerts (A and B) using the embedded statistical based method that was used in the clinical trial vs. the deep learning-based approach used in post-analysis. Panel C illustrates the flowchart of our novel post-analysis AI algorithm.

We developed a deep learning based approach that uses a convolutional denoising autoencoder to improve PPG signal quality for subsequent AF detection [27]. Employing this artificial intelligence algorithm successfully reduced the number of participants with false positives from ten to two participants (Figure 4). The total number of false positive alerts was also reduced from 35 to 6 (Figure 4).

## 4. Discussion

Several smartwatches are FDA cleared for AF detection and these devices may have particular value among older adults who survive an embolic stroke of undetermined source [31–33]. Older adults at highest risk for AF are also at highest risk for other arrhythmias (e.g., premature atrial beats, atrial ectopy, atrial tachycardia) and conditions (e.g., tremors) that may decrease the accuracy of wrist-based wearables for AF detection [34–38]. There have been reports of anxiety among smartwatch users who receive false AF alerts and studies are needed to better understand the performance and acceptability of wrist-based wearables for AF detection in older populations [39,40]. The use of smartwatches has not to date directly been associated with harms, but falsely abnormal results may be associated with anxiety and potentially have a negative impact on the psychological health of participants. The Screening for Atrial Fibrillation in the Elderly (SAFE) study was a multicenter trial that randomized several clinical practices to screening (systematic or opportunistic) vs. no screening for atrial fibrillation [41,42]. Anxiety scores were not significantly different between systematic and opportunistic AF screening arms. The study, however, did not collect any anxiety data points from participants in the no screening group, and no comparative analysis was possible between the screening and no screening groups. Using data from the Pulsewatch study, we sought to understand the connections between false AF alerts with patient reported outcomes that relate to overall patient well-being and healthcare utilization. We observed that false alerts occurred in 67% of participants who received any alert. This is consistent with other real-world studies involving smart watch users, including the Apple Heart study that demonstrated only 34% of the 450 participants with irregular pulse alerts on smartwatch had AF diagnosed on subsequent ECG patch monitor [18]. In our study, the most common cause of a false alert was a poor quality PPG signal. The embedded motion artifact detection algorithm in the smartwatch was based on statistical features and a threshold value derived from the time-frequency representation using the preliminary data collected from 37 subjects wearing a non-commercially-available Samsung smartwatch, Simband, in a clinical environment [21]. The data collection duration was 14 minutes and the protocol involved subjects performing limited daily activities. The accuracy of the AF detection algorithm was constrained by the narrow memory of the smarwatch, modest training dataset and the maximization of near real-time calculations. The second cause for false alerts was non-AF arrythmias, such as sinus arrythmia, premature atrial complexes (PACs) and premature ventricular complexes (PVCs). Our work is consistent with previous findings of Bashar and colleagues that false positives are related to noise artifact, PAC and PVC [21]. While we tried to account for PACs and PVCs, the training data contained only 6 subjects with these rhythms [43]. More importantly, there were only 46 segments to train PAC/PVC from these subjects which is not sufficient to account for different dynamics of these rhythms. Hence, it is not surprising that our embedded algorithm was not able to accurately differentiate PAC/PVC from AF beats. When we applied our deep learning approach for AF detection, we reduced the number of false positive alerts by 83%. Our deep learning approach developed for offline analysis was trained with 60 times more segments than the number used in our embedded rule-based AF detection [44]. As the results showed successful reduction of the number of false positive alerts using deep learning, we concluded that having a sufficiently large training dataset is paramount to account for various complicated rhythms for both deep learning and statistical rule-based AF detection algorithms. Although we did not identify physiological, psychosocial, socio-demographic, or other characteristics associated with receipt of a false alert in our sample of older stroke survivors, we observed that health-related quality of life and confidence in symptom management decreased significantly in participants receiving false AF alerts. Participants who received false alerts might have reported worsened quality of life and lower confidence in their disease management due to physical manifestations of PACs or PVCs, albeit a rather low proportion of false alerts was attributed to those arrhythmias (less than 11 out of 35 false alerts, Figure 3). It is also possible that these alerts contributed to heightened awareness or worry independent of their cardiac rhythm. An alternative explanation might be that participants who received a false alert may have experienced AF outside of the monitoring window (for example at nighttime) and therefore their symptoms were attributable to undetected cardiac arrhythmias. Consistent with findings of decreased physical health perceptions, participants who received false alerts also reported decreased confidence in chronic symptom management. Participants who receive alerts may feel overwhelmed and lose self-confidence as they do not know how to manage alerts and have little power to stop the alarms. Of note, there was no change in self-reported medication adherence among those who received a false alert. There appears to be a dose-response relationship with increasing number of false alerts and decreasing self-reported physical health and self-reported confidence in chronic symptom management. In comparison to those who receive ≤ 2 false alerts, those who received > 2 false alerts reported greater reduction in perceived physical health and confidence in chronic symptom management during study period. Results suggest a potential threshold after which alerts can cause significant decline in a patient’s perceived wellbeing. Our findings are consistent with previous reports that false positive alerts are associated with negative short-term psychosocial consequences, affecting self-perception, and decreasing short-term quality of life [45–48]. Here, we address several gaps in our understanding of the impact of false alerts in a population wearing contemporaneous watches and patch monitors. Consistent with our previously published work, we found that smartwatch alerts for AF (both true and false positives) cause significant decline in self-reported physical health in a dose-dependent manner [48]. Our findings suggest that clinicians should consider the stress and potential adverse impact of false alerts before recommending commercial wearables for AF detection and should educate patients about what to do should they receive an AF alert. Previously, Ding *et al.* found that health care providers report difficulty interpreting tracings from commercial wearables and a lack of knowledge about the appropriate workup of patients with a possible AF alert [49]. Wang *et al.* reported that healthcare utilization, including ablation procedures, was higher among patients with AF and wearables as compared to patients with AF and no wearables, even when controlling for their baseline heart rate [7]. Further studies are needed to optimize AF detection algorithms for long-term monitoring in older populations to minimize the potential negative impact of false positives alerts. Our findings suggest that clinicians should educate their patients about the limitations of commercial wearables and discuss the potential risks and benefits of these devices. More real-world studies, like HEARTLINE (NCT04276441), are needed to examine the clinical impact of smartwatch prescription for patients with or at risk for AF. Finally, society guidelines for clinicians and patient-education materials are needed to help healthcare providers and their patients navigate the increasingly complex area of mobile health and arrhythmia surveillance.

## 5. Strength and Limitations

Our study has multiple strengths. It is a multifaceted randomized clinical trial to evaluate the accuracy and health behavior impact of wearables for AF detection in stroke survivors. Participants in this study are well-defined with respect to sociodemographic, clinical, and psychosocial characteristics. We applied validated instruments including PEPPI, GAD-7, CHAI, and SF-12 at two time-points to examine changes in patient-physician interaction, anxiety, patient activation, and health-related quality of life among participants, increasing the generalizability and likely reproducibility of our study findings. There are some limitations that should be considered when interpreting our findings. Our sample size is modest with relatively short follow-up. It is not designed or powered to evaluate the effects of false alerts on measured outcomes in long-term use of smartwatches for AF detection. Furthermore, our cohort is relatively homogeneous with respect to race, ethnicity, and socioeconomic status, and only includes stroke survivors, limiting the generalizability of our findings to populations not represented in our study cohort. Finally, we only examined the impact of alerts from one smartwatch-smartphone system and it is possible that other alert systems do not elicit the same response from users.

## 6. Conclusions

We observed that a modest proportion of individuals received a false AF alert in our randomized trial of older stroke survivors over a two-week period. We noted a significant, dose-dependent negative impact of false AF alerts on both health-related quality of life and chronic disease self-management. A promising approach to avoid negative impact of false alerts is to employ artificial intelligence driven algorithms to improve accuracy. Further study is warranted to improve AI algorithm of commercial wearables for AF detection and new tools are needed to help guide healthcare providers, patients, and caregivers to make informed decisions about smartwatch use given the potential adverse impact of false alerts in this population.

## Supplementary Material

supply

## Figures and Tables

**Table 1:** Baseline characteristics of participants who received false alerts compared to those who received no alerts. Abbreviations: BMI- Body Mass Index; BP- Blood Pressure; COPD- Chronic Obstructive Pulmonary Disease; HR- Heart Rate.

Socio-demographics	Total (n=80)	Yes (n=10)	No (n=70)	P- value
Age, mean, years (SD)	64.3 (9.1)	64.9 (9.9)	64.2 (9.0)	0.82
Female sex (%)	34 (42.5)	4 (40.0)	30 (42.9)	1
Race (%)				
White	69 (86.3)	9 (90.0)	60 (85.7)	0.33
Other	11 (13.7)	1 (10.0)	10 (14.3)	
Married/Living as married (%)	54 (68.4)	7 (70.0)	47 (68.1)	0.2
Education (%)				
Less than high school	2 (2.5)	0 (0.00)	2 (2.9)	0.82
High school degree or some college	36 (45.6)	6 (60.0)	30 (43.5)	
College degree	18 (22.8)	2 (20.0)	16 (23.2)	
Post-graduate studies/degree	23 (29.1)	2 (20.0)	21 (30.4)	
Income (%)				
Less than 50,000$ annually	25 (33.8)	4 (40.0)	21 (32.8)	0.96
50,000 – 99,999$ annually	23 (31.1)	3 (30.0)	20 (31.3)	
Over 100,000$ annually	26 (35.1)	3 (30.0)	23 (35.9)	
Physiologic parameters				
BMI, mean (SD)	32.4 (22.3)	31.8 (8.0)	32.5 (23.7)	0.86
Systolic BP, mean (SD)	130.4 (15.3)	134.5 (16.9)	129.9 (15.1)	0.37
Diastolic BP, mean (SD)	76.5 (8.0)	79.8 (6.5)	76.0 (8.1)	0.16
HR, mean (SD)	72.8 (15.0)	71.8 (22.9)	72.9 (13.7)	0.88
Past Medical History (%)				
Vascular Disease	20 (25.0)	3 (30.0)	17 (24.3)	0.7
Valvular Disease	8 (10.0)	0 (0)	8 (11.4)	0.59
Diabetes Mellitus	19 (23.8)	1 (10.0)	18 (25.7)	0.44
COPD	6 (7.5)	0 (0.0)	6 (8.6)	1
Renal disease	3 (3.8)	1 (10.0)	2 (2.9)	0.33
Major bleeding event or predisposition to bleeding	5 (6.3)	1 (10.0)	4 (5.7)	0.5
Congestive Heart Failure	5 (6.3)	1 (10.0)	4 (5.7)	0.5
Essential Hypertension	62 (77.5)	8 (80.0)	54 (77.1)	1
Obstructive Sleep Apnea	22 (27.5)	2 (20.0)	20 (28.6)	0.72
Prior myocardial infarction	14 (17.5)	2 (20.0)	12 (17.1)	1
Hyperlipidemia	67 (83.8)	9 (90.0)	58 (82.9)	1
Stroke History (%)				
Stroke	65 (81.3)	8 (80.0)	57 (81.4)	1
TIA	22 (27.5)	4 (40.0)	18 (25.7)	0.45
Residual Neurologic Deficits	27 (33.8)	2 (20.0)	25 (35.7)	0.6
Medication use (%)				
Anticoagulants	10 (12.5)	1 (10.0)	9 (12.9)	1
Antiplatelets	70 (87.5)	9 (90.0)	61 (87.1)	1
Antihypertensives	45 (56.3)	3 (30.0)	42 (60.0)	0.09
Anti-arrhythmic medications	2 (2.5)	0 (0.0)	2 (2.9)	1
Beta blockers	37 (46.3)	5 (50.0)	32 (45.7)	1
Statins	73 (91.3)	10 (100.0)	63 (90.0)	0.59

**Table 2:** Baseline psychosocial and technology engagement characteristics participants who received false alerts compared to those who received no alerts.

Characteristics	Receiving Alerts			
Socio-demographics	Total (n=80)	Yes (n=10)	No (n=70)	P- value
**Psychosocial characteristics (%)**				
Cognitive impairment	22 (28.2)	3 (30.0)	19 (27.9)	1
Social isolation	9 (11.3)	0 (0)	9 (12.9)	0.59
>8 alcoholic drinks per week	6 (7.5)	0 (0)	6 (8.6)	1
Depressive symptoms				
Minimal	44 (55.0)	4 (40.0)	40 (57.1)	0.49
Mild	24 (30.0)	4 (40.0)	20 (28.6)	
Moderate	8 (10.0)	2 (20.0)	6 (8.6)	
Moderately severe	3 (3.7)	0 (0)	3 (4.3)	
Severe	1 (1.3)	0 (0)	1 (1.4)	
Anxiety Symptoms				
Minimal	55 (69.6)	8 (80.0)	47 (68.1)	0.9
Mild	15 (19.0)	1 (10.0)	14 (20.3)	
Moderate	7 (8.9)	1 (10.0)	6 (8.7)	
Severe	2 (2.5)	0 (0)	2 (2.9)	
Patient activation				
Low	30 (38.0)	4 (40.0)	26 (37.7)	0.33
Medium	36 (45.6)	6 (60.0)	30 (43.5)	
High	13 (16.4)	0 (0)	13 (18.8)	
**Technology engagement (%)**				
Device Ownership				
Smartphone	68 (85.0)	10 (100.0)	58 (82.9)	0.34
Smartwatch	20 (25.0)	2 (20.0)	18 (25.7)	1
App use frequency				
Daily	50 (69.4)	6 (60.0)	44 (70.9)	0.39
A few days a week	11 (15.3)	3 (30.0)	8 (12.9)	
At least once a week	4 (5.5)	0 (0)	4 (6.5)	
Less than once a week	2 (2.8)	0 (0)	2 (3.2)	
Once a month	2 (2.8)	1 (10.0)	1 (1.6)	
Never	3 (4.2)	0 (0)	3 (4.8)	

**Table 3:** Health behavior impact of false AF alerts.

False alerts (N=10) vs. no alerts (N=70)	ß Estimate (SE)	P value
Generalized Anxiety Disorder-7 score	−1.11 (1.28)	0.39
Consumer Health Activation Index score	−6.04 (4.14)	0.15
Physical Health Short Form-12 survey	−7.53 (3.20)	**0.02**
Mental Health Short Form-12 survey	1.14 (2.65)	0.67
Chronic Symptom Management Self-efficacy	−8.32 (2.81)	**0.004**
Medication Adherence	−1.06 (0.99)	0.29

* higher GAD7 indicates poorer psychosocial outcome (anxiety), while higher CHAI or SF-12 indicates better psychosocial outcomes (patient activation and self-reported health respectively).

**Table 4:** Health behavior impact of false AF alerts for ≤ 2 or >2 alerts compared to no alerts.

	1 or 2 alerts (N=5)		>2 alerts (N=5)	
	ß Estimate (SE)	p- value	Estimate (SE)	p- value
Generalized Anxiety Disorder-7 score	−1.80 (1.74)	0.3	−0.39 (1.76)	0.83
Consumer Health Activation Index score	−6.44 (5.66)	0.26	−5.64 (5.66)	0.32
Physical Health SF-12 PCS	−0.99 (4.24)	0.82	−14.08(4.24)	**0.001**
Mental Health SF-12 MCS	−0.01 (3.62)	0.99	2.28 (3.62)	0.53
Chronic Symptom Management	−4.32 (3.80)	0.26	−12.32(3.80)	**0.002**
Medication Adherence	−0.86 (1.35)	0.53	−1.26 (1.35)	0.35

* higher GAD7 indicates poorer psychosocial outcome (anxiety), while higher CHAI or SF-12 indicates better psychosocial outcomes (patient activation and self-reported health respectively).

## Data Availability

Data from the Pulsewatch study will be made available upon request, subject to approval of the University of Massachusetts Chan Medical School Institutional Review Board. All questions can be addressed to khanh-van.tran@umassmemorial.org.

## References

[1] Lippi G, Sanchis-Gomar F, Cervellin G. Global epidemiology of atrial fibrillation: An increasing epidemic and public health challenge. Int J Stroke 16 (2021): 217–221. 31955707 10.1177/1747493019897870

[2] Kernan WN, Ovbiagele B, Black HR, Guidelines for the prevention of stroke in patients with stroke and transient ischemic attack: a guideline for healthcare professionals from the American Heart Association/American Stroke Association. Stroke 45 (2014): 2160–2236. 24788967 10.1161/STR.0000000000000024

[3] Yaghi S, Kamel H. Stratifying Stroke Risk in Atrial Fibrillation: Beyond Clinical Risk Scores. Stroke 48 (2017): 2665–2670. 28916670 10.1161/STROKEAHA.117.017084 PMC5679200

[4] Lubitz SA, Yin X, McManus DD, Stroke as the Initial Manifestation of Atrial Fibrillation: The Framingham Heart Study. Stroke 48(2017): 490–492. 28082669 10.1161/STROKEAHA.116.015071 PMC5262530

[5] Force USPST Curry SJ, Krist AH, Screening for Atrial Fibrillation: US Preventive Services Task Force Recommendation Statement. JAMA 327 (2022): 360–367. 35076659 10.1001/jama.2021.23732

[6] Kleindorfer DO, Towfighi A, Chaturvedi S, 2021 Guideline for the Prevention of Stroke in Patients With Stroke and Transient Ischemic Attack: A Guideline From the American Heart Association/American Stroke Association. Stroke 52 (2021): e364–e467. 34024117 10.1161/STR.0000000000000375

[7] Wang L, Nielsen K, Goldberg J, Association of Wearable Device Use With Pulse Rate and Health Care Use in Adults With Atrial Fibrillation. JAMA Netw Open 4 (2021): e215821. 34042996 10.1001/jamanetworkopen.2021.5821 PMC8160588

[8] Rosenberg MA, Samuel M, Thosani A, Use of a noninvasive continuous monitoring device in the management of atrial fibrillation: a pilot study. Pacing Clin Electrophysiol 36 (2013): 328–333. 23240827 10.1111/pace.12053 PMC3618372

[9] Derkac WM, Finkelmeier JR, Horgan DJ, Diagnostic yield of asymptomatic arrhythmias detected by mobile cardiac outpatient telemetry and autotrigger looping event cardiac monitors. J Cardiovasc Electrophysiol 28 (2017): 1475–1478. 28940881 10.1111/jce.13342

[10] Adam BTM, Mehta P, Ghanbari H. Real-world performance of atrial fibrillation detection from wearable patient ECG monitoring using deep learning. Heart rhythm (2019).

[11] Ko D, Dai Q, Flynn DB, Meta-Analysis of Randomized Clinical Trials Comparing the Impact of Implantable Loop Recorder Versus Usual Care After Ischemic Stroke for Detection of Atrial Fibrillation and Stroke Risk. Am J Cardiol 162 (2022): 100–104. 34756594 10.1016/j.amjcard.2021.09.013 PMC8678332

[12] Palmer AJ, Valentine WJ, Roze S, Overview of costs of stroke from published, incidence-based studies spanning 16 industrialized countries. Curr Med Res Opin 21 (2005): 19–26. 15881472 10.1185/030079904x17992

[13] Vogels EA. About one-in-five Americans use a smart watch or fitness tracker. (2020).

[14] Tison GH, Sanchez JM, Ballinger B, Passive Detection of Atrial Fibrillation Using a Commercially Available Smartwatch. JAMA Cardiol 3 (2018): 409–416. 29562087 10.1001/jamacardio.2018.0136 PMC5875390

[15] Bumgarner JM, Lambert CT, Hussein AA, Smartwatch Algorithm for Automated Detection of Atrial Fibrillation. J Am Coll Cardiol 71 (2018): 2381–2388. 29535065 10.1016/j.jacc.2018.03.003

[16] Nemati S, Ghassemi MM, Ambai V, Monitoring and detecting atrial fibrillation using wearable technology. Annu Int Conf IEEE Eng Med Biol Soc 2016 (2016): 3394–3397. 10.1109/EMBC.2016.7591456 28269032

[17] Guo Y, Wang H, Zhang H, Mobile Photoplethysmographic Technology to Detect Atrial Fibrillation. J Am Coll Cardiol 74 (2019): 2365–2375. 31487545 10.1016/j.jacc.2019.08.019

[18] Perez MV, Mahaffey KW, Hedlin H, Large-Scale Assessment of a Smartwatch to Identify Atrial Fibrillation. N Engl J Med 381 (2019): 1909–1917. 31722151 10.1056/NEJMoa1901183 PMC8112605

[19] Brandes A, Stavrakis S, Freedman B, Consumer-Led Screening for Atrial Fibrillation: Frontier Review of the AF-SCREEN International Collaboration. Circulation 146 (2022): 1461–1474. 36343103 10.1161/CIRCULATIONAHA.121.058911 PMC9673231

[20] Dickson EL, Ding EY, Saczynski JS, Smartwatch monitoring for atrial fibrillation after stroke-The Pulsewatch Study: Protocol for a multiphase randomized controlled trial. Cardiovasc Digit Health J 2 (2021): 231–241. 35265913 10.1016/j.cvdhj.2021.07.002 PMC8890084

[21] Bashar SK, Han D, Hajeb-Mohammadalipour S, Atrial Fibrillation Detection from Wrist Photoplethysmography Signals Using Smartwatches. Sci Rep 9 (2019): 15054. 31636284 10.1038/s41598-019-49092-2 PMC6803677

[22] Spitzer RL, Kroenke K, Williams JB, A brief measure for assessing generalized anxiety disorder: the GAD-7. Arch Intern Med 166 (2006): 1092–1097. 16717171 10.1001/archinte.166.10.1092

[23] Wolf MS, Smith SG, Pandit AU, Development and Validation of the Consumer Health Activation Index. Med Decis Making 38 (2018): 334–343. 29436308 10.1177/0272989X17753392 PMC6329370

[24] Huo T, Guo Y, Shenkman E, Assessing the reliability of the short form 12 (SF-12) health survey in adults with mental health conditions: a report from the wellness incentive and navigation (WIN) study. Health Qual Life Outcomes 16 (2018): 34. 29439718 10.1186/s12955-018-0858-2 PMC5811954

[25] Sarkar U, Fisher L, Schillinger D. Is self-efficacy associated with diabetes selfmanagement across race/ethnicity and health literacy? Diabetes Care 29 (2006): 823–829. 16567822 10.2337/diacare.29.04.06.dc05-1615

[26] Han D, Pulsewatch: A Smartwatch System Designed with Stroke Survivors for Continuous Monitoring of Atrial Fibrillation in Older Adults after Stroke or Transient Ischemic Attack Journal of Medical Internet Research In submission (2022).

[27] Mohagheghian F, Atrial Fibrillation Detection on Reconstructed Photoplethysmography Signals Collected from a Smart Watch using Denoising Autoencoder Expert Systems With Applications Manuscript submitted for publication (2022).

[28] Chiang HT, Hsieh Y, Fu S, Noise Reduction in ECG Signals Using Fully Convolutional Denoising Autoencoders. IEEE Access 7 (2019): 60806–60813.

[29] Singh P, Sharma A. Attention-Based Convolutional Denoising Autoencoder for Two- Lead ECG Denoising and Arrhythmia Classification. IEEE Transactions on Instrumentation and Measurement 71 (2022): 1–10.

[30] Torres-Soto J EA A. Multi-task deep learning for cardiac rhythm detection in wearable devices. npj Digital Medicine 3 (2020). 10.1038/s41746-020-00320-4 PMC7481177 32964139

[31] Lubitz SA, Faranesh AZ, Atlas SJ, Rationale and design of a large population study to validate software for the assessment of atrial fibrillation from data acquired by a consumer tracker or smartwatch: The Fitbit heart study. Am Heart J 238 (2021): 16–26. 33865810 10.1016/j.ahj.2021.04.003

[32] Saghir N, Aggarwal A, Soneji N, A comparison of manual electrocardiographic interval and waveform analysis in lead 1 of 12-lead ECG and Apple Watch ECG: A validation study. Cardiovasc Digit Health J 1 (2020): 30–36. 35265871 10.1016/j.cvdhj.2020.07.002 PMC8890353

[33] Seshadri DR, Bittel B, Browsky D, Accuracy of Apple Watch for Detection of Atrial Fibrillation. Circulation 141 (2020): 702–703. 32091929 10.1161/CIRCULATIONAHA.119.044126

[34] Perino AC, Gummidipundi SE, Lee J, Arrhythmias Other Than Atrial Fibrillation in Those With an Irregular Pulse Detected With a Smartwatch: Findings From the Apple Heart Study. Circ Arrhythm Electrophysiol 14 (2021): e010063. 34565178 10.1161/CIRCEP.121.010063

[35] Strik M, Ploux S, Ramirez FD, Smartwatch-based detection of cardiac arrhythmias: Beyond the differentiation between sinus rhythm and atrial fibrillation. Heart Rhythm 18 (2021): 1524–1532. 34147700 10.1016/j.hrthm.2021.06.1176

[36] Caillol T, Strik M, Ramirez ED, Accuracy of a Smartwatch-Derived ECG for Diagnosing Bradyarrhythmias, Tachyarrhythmias, and Cardiac Ischemia. Circ Arrhythm Electrophysiol 14 (2021): e009260. 33441002 10.1161/CIRCEP.120.009260

[37] Osman W, Hanson M, Baranchuk A. Pseudo-Ventricular Tachycardia, Pseudo-Atrial Fibrillation, and Pseudo-Atrial Flutter in a Patient With Parkinson Disease: Two’s Company, Three’s a Crowd. JAMA Intern Med 179 (2019): 824–826. 31009038 10.1001/jamainternmed.2019.0746

[38] Hwang WJ, Chen JY, Sung PS, Parkinsonian tremor-induced electrocardiographic artifacts mimicking atrial flutter/fibrillation or ventricular tachycardia. Int J Cardiol 173 (2014): 597–600. 24698226 10.1016/j.ijcard.2014.03.090

[39] Jonas DE, Kahwati LC, Yun JDY, Screening for Atrial Fibrillation With Electrocardiography: Evidence Report and Systematic Review for the US Preventive Services Task Force. JAMA 320 (2018): 485–498. 30088015 10.1001/jama.2018.4190

[40] Boriani G, Schnabel RB, Healey JS, Consumer-led screening for atrial fibrillation using consumer-facing wearables, devices and apps: A survey of health care professionals by AF-SCREEN international collaboration. Eur J Intern Med 82 (2020): 97–104. 32933842 10.1016/j.ejim.2020.09.005

[41] Fitzmaurice DA, Richard Hobbs FD, Jowett S, Screening versus routine practice in detection of atrial fibrillation in patients aged 65 or over: cluster randomised controlled trial. BMJ 335 (2007): 383. 17673732 10.1136/bmj.39280.660567.55 PMC1952508

[42] Fitzmaurice DA, McCahon D, Baker J, Is screening for AF worthwhile? Stroke risk in a screened population from the SAFE study. Fam Pract 31 (2014): 298–302. 24728774 10.1093/fampra/cmu011

[43] Han D, Bashar SK, Mohagheghian F, Premature Atrial and Ventricular Contraction Detection using Photoplethysmographic Data from a Smartwatch. Sensors (Basel) 20 (2020). 10.3390/s20195683 PMC7582300 33028000

[44] Mohagheghian F, Atrial Fibrillation Detection on Reconstructed Photoplethysmography Signals Collected from a Smart watch using Denoising Autoencoder IEEE J Biomed Health Inform In revision (2022).

[45] Rasmussen JF, Siersma V, Malmqvist J, Psychosocial consequences of false positives in the Danish Lung Cancer CT Screening Trial: a nested matched cohort study. BMJ Open 10 (2020): e034682. 10.1136/bmjopen-2019-034682 PMC7279658 32503869

[46] Toft EL, Kaae SE, Malmqvist J, Psychosocial consequences of receiving false-positive colorectal cancer screening results: a qualitative study. Scand J Prim Health Care 37 (2019): 145–154. 31079520 10.1080/02813432.2019.1608040 PMC6566584

[47] Hafslund B, Espehaug B, Nortvedt MW. Effects of false-positive results in a breast screening program on anxiety, depression and health-related quality of life. Cancer Nurs 35 (2012): E26–34. 22067696 10.1097/NCC.0b013e3182341ddb

[48] Filippaios A, Tran KT, Mehawej J, Psychosocial measures in relation to smartwatch alerts for atrial fibrillation detection. Cardiovasc Digit Health J 3 (2022): 198–200. 36310684 10.1016/j.cvdhj.2022.07.069 PMC9596300

[49] Ding EY, Svennberg E, Wurster C, Survey of current perspectives on consumer-available digital health devices for detecting atrial fibrillation. Cardiovasc Digit Health J 1 (2020): 21–29. 32924024 10.1016/j.cvdhj.2020.06.002 PMC7452829

