# Supplementary material for: False Atrial Fibrillation Alerts from Smartwatches are Associated with Decreased Perceived Physical Well-being and Confidence in Chronic Symptoms Management
Source: Cardiol Cardiovasc Med. Author manuscript; Available in PMC 2023 Jul 20. (PMC10358285; doi:10.26502/fccm.92920314)
Supplement: supply [file NIHMS1888653-supplement-supply.docx]

**Title:** **False Atrial Fibrillation Alerts from Smartwatches are Associated with Decreased Perceived Physical Well-being and Confidence in Chronic Symptoms Management**

Author list: Khanh-Van Tran ^1#*^, Andreas Filippaios ^1#^, Kamran Noorishirazi ^1^, Eric Ding ^1,2^, Dong Han^3^, Fahimeh Mohagheghian^3^, Qiying Dai^4^, Jordy Mehawej ^1,2^, Ziyue Wang ^1^, Darleen Lessard ^1,2^, Edith Mensah Otabil ^1^, Alex Hamel ^1^, Tenes Paul^1^, Matthew F. Gottbrecht^1^, Timothy P. Fitzgibbons^1^, Jane Saczynski^5^, Ki H. Chon^4^, David D. McManus ^1,3^

^1^ Division of Cardiovascular Medicine, Department of Medicine, University of Massachusetts Chan Medical School, 55 Lake Avenue North, Worcester, MA 01655, USA.

^2^ Department of Population and Quantitative Health Sciences, University of Massachusetts Chan Medical School, 55 Lake Avenue North, Worcester, MA 01655, USA.

^3^ Department of Biomedical Engineering, University of Connecticut, 260 Glenbrook Road, Storrs, CT 06269, USA

^4^ Division of Cardiovascular Medicine, Department of Medicine, Saint Vincent Hospital, 123 Summer Street, Worcester, MA 01608, USA.

^5^ Department of Pharmacy and Health Systems Sciences, Northeastern University, Boston, Massachusetts.

^#^ Equal contributions

**Supplemental Information**

**Supplemental Table 1.** Baseline characteristics of participants prescribed smartwatch for AF detection who received any alerts vs no alerts

| **Characteristics** | **Receiving Alerts** | | |
| --- | --- | --- | --- |
| **Socio-demographics** | **Yes (n=15)** | **No (n=70)** | **P-value** |
| Age, mean, years (SD) | 67.8 (9.5) | 64.2 (9.0) | 0.17 |
| Female sex (%) | 5 (33.3) | 30 (42.9) | 0.57 |
| Race (%) |  |  |  |
| White | 13 (86.7) | 60 (85.7) | 0.43 |
| Other | 2 (13.3) | 10 (14.3) |  |
| Married/Living as married (%) | 11 (73.3) | 47 (68.1) | 0.07 |
| Education (%) |  |  |  |
| Less than high school | 1 (6.7) | 2 (2.9) | 0.69 |
| High school degree or some college | 7 (46.6) | 30 (43.5) |  |
| College degree | 3 (20.0) | 16 (23.2) |  |
| Post-graduate studies/degree | 4 (26.7) | 21 (30.4) |  |
| Income (%) |  |  |  |
| Less than 50,000$ annually | 6 (40.0) | 21 (32.8) | 0.95 |
| 50,000 – 99,999$ annually | 5 (33.3) | 20 (31.3) |  |
| Over 100,000$ annually | 4 (26.7) | 23 (35.9) |  |
| **Physiologic parameters** |  |  |  |
| BMI, mean (SD) | 30.9 (6.6) | 32.5 (23.7) | 0.64 |
| Systolic BP, mean (SD) | 139.5 (19.7) | 129.9 (15.1) | 0.04 |
| Diastolic BP, mean (SD) | 78.4 (7.7) | 76.0 (8.1) | 0.30 |
| HR, mean (SD) | 71.4 (19.4) | 72.9 (13.7) | 0.72 |
| **Past Medical History (%)** |  |  |  |
| Vascular Disease | 4 (26.7) | 17 (24.3) | 1.00 |
| Valvular Disease | 0 (0) | 8 (11.4) | 0.34 |
| Diabetes Mellitus | 5 (33.3) | 18 (25.7) | 0.54 |
| COPD | 1 (6.7) | 6 (8.6) | 1.00 |
| Renal disease | 1 (6.7) | 2 (2.9) | 0.45 |
| Major bleeding event or predisposition to bleeding | 1 (6.7) | 4 (5.7) | 1.00 |
| Congestive Heart Failure | 2 (13.3) | 4 (5.7) | 0.29 |
| Essential Hypertension | 11 (73.3) | 54 (77.1) | 0.74 |
| Obstructive Sleep Apnea | 3 (20.0) | 20 (28.6) | 0.75 |
| Prior myocardial infarction | 3 (20.0) | 12 (17.1) | 0.72 |
| Hyperlipidemia | 14 (93.3) | 58 (82.9) | 0.45 |
|  |  |  |  |
| **Stroke History (%)** |  |  |  |
| Stroke | 11 (73.3) | 57 (81.4) | 0.49 |
| TIA | 6 (40.0) | 18 (25.7) | 0.34 |
| Residual Neurologic Deficits | 2 (13.3) | 25 (35.7) | 0.12 |
| **Medication use (%)** |  |  |  |
| Anticoagulants | 1 (6.7) | 9 (12.9) | 0.68 |
| Antiplatelets | 14 (93.3) | 61 (87.1) | 0.68 |
| Antihypertensives | 6 (40.0) | 42 (60.0) | 0.25 |
| Anti-arrhythmic medications | 0 (0) | 2 (2.9) | 1.00 |
| Beta blockers | 6 (40.0) | 32 (45.7) | 0.78 |
| Statins | 15 (100.0) | 63 (90.0) | 0.34 |
| **Psychosocial characteristics (%)** |  |  |  |
| Cognitive impairment | 5 (33.3) | 19 (27.9) | 0.76 |
| Social isolation | 1 (6.7) | 9 (12.9) | 0.68 |
| >8 alcoholic drinks per week | 1 (6.7) | 6 (8.6) | 1.00 |
| Depressive symptoms |  |  |  |
| Minimal | 8 (53.3) | 40 (57.1) | 0.89 |
| Mild | 5 (33.3) | 20 (28.6) |  |
| Moderate | 2 (13.3) | 6 (8.6) |  |
| Moderately severe | 0 (0) | 3 (4.3) |  |
| Severe | 0 (0) | 1 (1.4) |  |
| Anxiety Symptoms |  |  |  |
| Minimal | 12 (80.0) | 47 (68.1) | 0.93 |
| Mild | 2 (13.3) | 14 (20.3) |  |
| Moderate | 1 (6.7) | 6 (8.7) |  |
| Severe | 0 (0) | 2 (2.9) |  |
| Patient activation |  |  |  |
| Low | 5 (35.7) | 26 (37.7) | 0.54 |
| Medium | 8 (57.1) | 30 (43.5) |  |
| High | 1 (7.1) | 13 (18.8) |  |
| **Technology engagement (%)** |  |  |  |
| Device Ownership |  |  |  |
| Smartphone | 13 (86.7) | 58 (82.9) | 1.00 |
| Smartwatch | 3 (20.0) | 18 (25.7) | 0.75 |
| App use frequency |  |  |  |
| Daily | 8 (57.1) | 44 (71.0) | 0.40 |
| A few days a week | 4 (28.6) | 8 (12.9) |  |
| At least once a week | 1 (7.1) | 4 (6.5) |  |
| Less than once a week | 0 (0) | 2 (3.2) |  |
| Once a month | 1 (7.1) | 1 (1.6) |  |
| Never | 0 (0) | 3 (4.8) |  |
| Abbreviations: BMI: Body Mass Index; BP: Blood Pressure; COPD: Chronic Obstructive Pulmonary Disease; HR: Heart Rate | | | |

**Supplemental Table 2.** Relations between any AF alerts and survey scores among Pulsewatch participants

| **Received alerts (N=15) vs. no alerts (N=70)** | **Beta-Estimate (SE)** | **P value** |
| --- | --- | --- |
| Generalized Anxiety Disorder-7 score | -1.54 (1.06) | 0.15 |
| Consumer Health Activation Index score | -4.21 (3.54) | 0.24 |
| Physical Health Short Form-12 survey | -5.77 (2.74) | **0.04** |
| Mental Health Short Form-12 survey | 2.66 (2.20) | 0.23 |
| Chronic Symptom Management Self-efficacy | -6.08 (2.52) | **0.02** |
| Medication Adherence | -1.00 (0.81) | 0.22 |

**Supplemental Table 3.** Relations of 1 or 2 vs. >2 AF alerts compared to no alerts on survey scores among Pulsewatch participants

|  | | | | |
| --- | --- | --- | --- | --- |
|  | **1 or 2 alerts (N=7)** | | **>2 alerts (N=8)** | |
|  | **Beta Estimate (SE)** | **p-value** | **Beta Estimate (SE)** | **p-value** |
| Generalized Anxiety Disorder-7 score | -1.50 (1.47) | 0.31 | -1.58 (1.39) | 0.26 |
| Consumer Health Activation Index score | -2.64 (4.89) | 0.59 | -5.63 (4.66) | 0.23 |
| Physical Health SF-12 PCS | -3.50 (3.82) | 0.36 | -7.73 (3.57) | **0.03** |
| Mental Health SF-12 MCS | 0.85 (3.07) | 0.78 | 4.21 (2.86) | 0.14 |
| Chronic Symptom Management | -1.85 (3.45) | 0.59 | -9.79 (3.25) | **0.003** |
| Medication Adherence | -0.67 (1.12) | 0.55 | -1.29 (1.07) | 0.23 |

**Supplemental Table 4.** Baseline characteristics of Pulsewatch participants stratified by AF status and having received alerts vs no AF and receiving no alerts

| **Characteristics** | |  | | **Receiving Alerts** | | | |
| --- | --- | --- | --- | --- | --- | --- | --- |
| **Socio-demographics** | | **Total (n=74)** | **Yes (n=4)** | | **No (n=70)** | **P-value** |  |
| Age, mean, years (SD) | | 64.7 (9.1) | 73.0 (6.4) | | 64.2 (9.0) | 0.06 |  |
| Female sex (%) | | 31 (41.9) | 1 (25.0) | | 30 (42.9) | 0.64 |  |
| Race (%) | |  |  | |  |  |  |
| White | | 64 (86.5) | 4 (100.0) | | 60 (85.7) | 1.00 |  |
| Other | | 10 (13.5) | 0 (0) | | 10 (14.3) |  |  |
| Married/Living as married (%) | | 50 (68.5) | 3 (75.0) | | 47 (68.1) | 0.35 |  |
| Education (%) | |  |  | |  |  |  |
| Less than high school | | 3 (4.1) | 1 (25.0) | | 2 (2.9) | 0.12 |  |
| High school degree or some college | | 31 (42.5) | 1 (25.0) | | 30 (43.5) |  |  |
| College degree | | 16 (21.9) | 0 (0) | | 16 (23.2) |  |  |
| Post-graduate studies/degree | | 23 (31.5) | 2 (50.0) | | 21 (30.4) |  |  |
| Income (%) | |  |  | |  |  |  |
| Less than 50,000$ annually | | 22 (32.3) | 1 (25.0) | | 21 (32.8) | 0.51 |  |
| 50,000 – 99,999$ annually | | 22 (32.4) | 2 (50.0) | | 20 (31.3) |  |  |
| Over 100,000$ annually | | 24 (35.3) | 1 (25.0) | | 23 (35.9) |  |  |
| **Physiologic parameters** | |  |  | |  |  |  |
| BMI, mean (SD) | | 32.3 (23.0) | 29.6 (2.3) | | 32.5 (23.7) | 0.35 |  |
| Systolic BP, mean (SD) | | 130.8 (16.1) | 147.3 (26.2) | | 129.9 (15.1) | **0.04** |  |
| Diastolic BP, mean (SD) | | 76.1 (8.2) | 77.0 (10.9) | | 76.0 (8.1) | 0.82 |  |
| HR, mean (SD) | | 72.9 (13.5) | 71.8 (12.9) | | 72.9 (13.7) | 0.87 |  |
| **Past Medical History (%)** | |  |  | |  |  |  |
| Vascular Disease | | 18 (24.3) | 1 (25.0) | | 17 (24.3) | 1.00 |  |
| Valvular Disease | | 8 (10.8) | 0 (0) | | 8 (11.4) | 1.00 |  |
| Diabetes Mellitus | | 21 (28.4) | 3 (75.0) | | 18 (25.7) | 0.07 |  |
| COPD | | 7 (9.5) | 1 (25.0) | | 6 (8.6) | 0.33 |  |
| Renal disease | | 2 (2.7) | 0 (0) | | 2 (2.9) | 1.00 |  |
| Major bleeding event or predisposition to bleeding | | 4 (5.4) | 0 (0) | | 4 (5.7) | 1.00 |  |
| Congestive Heart Failure | | 5 (6.8) | 1 (25.0) | | 4 (5.7) | 0.25 |  |
| Essential Hypertension | | 56 (75.7) | 2 (50.0) | | 54 (77.1) | 0.25 |  |
| Obstructive Sleep Apnea | | 21 (28.4) | 1 (25.0) | | 20 (28.6) | 1.00 |  |
| Prior myocardial infarction | | 13 (17.6) | 1 (25.0) | | 12 (17.1) | 0.55 |  |
| Hyperlipidemia | | 62 (83.8) | 4 (100.0) | | 58 (82.9) | 1.00 |  |
|  | |  |  | |  |  |  |
| **Stroke History (%)** | |  |  | |  |  |  |
| Stroke | | 59 (79.7) | 2 (50.0) | | 57 (81.4) | 0.18 |  |
| TIA | | 20 (27.0) | 2 (50.0) | | 18 (25.7) | 0.29 |  |
| Residual Neurologic Deficits | | 25 (33.8) | 0 (0) | | 25 (35.7) | 0.43 |  |
| **Medication use (%)** | |  |  | |  |  |  |
| Anticoagulants | | 9 (12.2) | 0 (0) | | 9 (12.9) | 1.00 |  |
| Antiplatelets | | 65 (87.8) | 4 (100.0) | | 61 (87.1) | 1.00 |  |
| Antihypertensives | | 44 (59.5) | 2 (50.0) | | 42 (60.0) | 1.00 |  |
| Anti-arrhythmic medications | | 2 (2.7) | 0 (0) | | 2 (2.9) | 1.00 |  |
| Beta blockers | | 33 (44.6) | 1 (25.0) | | 32 (45.7) | 0.62 |  |
| Statins | | 67 (90.5) | 4 (100.0) | | 63 (90.0) | 1.00 |  |
| **Psychosocial characteristics (%)** | |  |  | |  |  |  |
| Cognitive impairment | | 20 (27.8) | 1 (25.0) | | 19 (27.9) | 1.00 |  |
| Social isolation | | 9 (12.2) | 0 (0) | | 9 (12.9) | 1.00 |  |
| >8 alcoholic drinks per week | | 7 (9.5) | 1 (25.0) | | 6 (8.6) | 0.33 |  |
| Depressive symptoms | |  |  | |  |  |  |
| Minimal | | 43 (58.1) | 3 (75.0) | | 40 (57.1) | 1.00 |  |
| Mild | | 21 (28.4) | 1 (25.0) | | 20 (28.6) |  |  |
| Moderate | | 6 (8.1) | 0 (0.0) | | 6 (8.6) |  |  |
| Moderately severe | | 3 (4.0) | 0 (0.0) | | 3 (4.3) |  |  |
| Severe | | 1 (1.4) | 0 (0) | | 1 (1.4) |  |  |
| Anxiety Symptoms | |  |  | |  |  |  |
| Minimal | | 50 (68.5) | 3 (75.0) | | 47 (68.1) | 1.00 |  |
| Mild | | 15 (20.6) | 1 (25.0) | | 14 (20.3) |  |  |
| Moderate | | 6 (8.2) | 0 (0) | | 6 (8.7) |  |  |
| Severe | | 2 (2.7) | 0 (0) | | 2 (2.9) |  |  |
| Patient activation | |  |  | |  |  |  |
| Low | | 27 (37.5) | 1 (33.3) | | 26 (37.7) | 0.80 |  |
| Medium | | 31 (43.1) | 1 (33.3) | | 30 (43.5) |  |  |
| High | | 14 (19.4) | 1 (33.3) | | 13 (18.8) |  |  |
| **Technology engagement (%)** | |  |  | |  |  |  |
| Device Ownership | |  |  | |  |  |  |
| Smartphone | | 60 (81.1) | 2 (50.0) | | 58 (82.9) | 0.16 |  |
| Smartwatch | | 18 (24.3) | 0 (0.0) | | 18 (25.7) | 0.57 |  |
| App use frequency | |  |  | |  |  |  |
| Daily | | 45 (69.2) | 1 (33.3) | | 44 (70.9) | 0.30 |  |
| A few days a week | | 9 (13.9) | 1 (33.3) | | 8 (12.9) |  |  |
| At least once a week | | 5 (7.7) | 1 (33.3 | | 4 (6.5) |  |  |
| Less than once a week | | 2 (3.1) | 0 (0) | | 2 (3.2) |  |  |
| Once a month | | 1 (1.5) | 0 (0) | | 1 (1.6) |  |  |
| Never | | 3 (4.6) | 0 (0) | | 3 (4.8) |  |  |
|  | Abbreviations: BMI: Body Mass Index; BP: Blood Pressure; COPD: Chronic Obstructive Pulmonary Disease; HR: Heart Rate | | | | | | |

**Supplemental Table 5.** Number of notifications, abnormalities, total alerts among the four trial participants with confirmed AF*

| Participant ID | **# of please stay still** | **# of abnormality** | **# of total alerts** |
| --- | --- | --- | --- |
| 005 | 15 | 3 | 18 |
| 017 | 18 | 5 | 23 |
| 075 | 13 | 4 | 17 |
| 082 | 1 | 0 | 1 |

 *watch data not available from the 5 participant with confirmed AF by patch and cardiologist.

**Supplemental Table 6**. Relations between AF alerts and survey scores among Pulsewatch participants who have confirmed AF and received alerts vs no AF and received no alerts

| **True alerts (N=4) vs. no alerts (N=70)** | **Beta-Estimate (SE)** | **P value** |
| --- | --- | --- |
| Generalized Anxiety Disorder-7 score | -2.00 (1.96) | 0.31 |
| Consumer Health Activation Index score | -1.89 (6.37) | 0.77 |
| Physical Health Short Form-12 survey | -3.56 (4.57) | 0.44 |
| Mental Health Short Form-12 survey | 5.68 (3.85) | 0.14 |
| Chronic Symptom Management Self-efficacy | -1.92 (4.04) | 0.64 |
| Medication Adherence | -0.88 (1.58) | 0.58 |
